# Prevalence of anopheline species and their *Plasmodium *infection status in epidemic-prone border areas of Bangladesh

**DOI:** 10.1186/1475-2875-9-15

**Published:** 2010-01-14

**Authors:** Mohammad Shafiul Alam, Md Gulam Musawwir Khan, Nurunnabi Chaudhury, Sharmina Deloer, Forida Nazib, A Mannan Bangali, Rashidul Haque

**Affiliations:** 1 Parasitology Laboratory, International Centre for Diarrhoeal Disease Research, Bangladesh (ICDDR,B), Dhaka-1212, Bangladesh; 2 Malaria and Parasitic Disease Control Unit, Directorate General of Health Services, Mohakhali, Dhaka-1212, Bangladesh; 3 World Health Organization, Dhaka, Bangladesh

## Abstract

**Background:**

Information related to malaria vectors is very limited in Bangladesh. In the changing environment and various *Anopheles *species may be incriminated and play role in the transmission cycle. This study was designed with an intention to identify anopheline species and possible malaria vectors in the border belt areas, where the malaria is endemic in Bangladesh.

**Methods:**

*Anopheles *mosquitoes were collected from three border belt areas (Lengura, Deorgachh and Matiranga) during the peak malaria transmission season (May to August). Three different methods were used: human landing catches, resting collecting by mouth aspirator and CDC light traps. Enzyme-linked immunosorbent assay (ELISA) was done to detect *Plasmodium falciparum*, *Plasmodium vivax*-210 and *Plasmodium vivax*-247 circumsporozoite proteins (CSP) from the collected female species.

**Results:**

A total of 634 female *Anopheles *mosquitoes belonging to 17 species were collected. *Anopheles vagus *(was the dominant species (18.6%) followed by *Anopheles nigerrimus *(14.5%) and *Anopheles philippinensis *(11.0%). Infection rate was found 2.6% within 622 mosquitoes tested with CSP-ELISA. Eight (1.3%) mosquitoes belonging to five species were positive for *P. falciparum*, seven (1.1%) mosquitoes belonging to five species were positive for *P. vivax *-210 and a single mosquito (0.2%) identified as *Anopheles maculatus *was positive for *P. vivax*-247. No mixed infection was found. Highest infection rate was found in *Anopheles karwari *(22.2%) followed by *An. maculatus *(14.3%) and *Anopheles barbirostris *(9.5%). Other positive species were *An. nigerrimus *(4.4%), *An. vagus *(4.3%), *Anopheles subpictus *(1.5%) and *An. philippinensis *(1.4%). *Anopheles vagus *and *An. philippinensis *were previously incriminated as malaria vector in Bangladesh. In contrast, *An. karwari*, *An. maculatus*, *An. barbirostris*, *An. nigerrimus *and *An. subpictus *had never previously been incriminated in Bangladesh.

**Conclusion:**

Findings of this study suggested that in absence of major malaria vectors there is a possibility that other *Anopheles *species may have been playing role in malaria transmission in Bangladesh. Therefore, further studies are required with the positive mosquito species found in this study to investigate their possible role in malaria transmission in Bangladesh.

## Background

Throughout the world, there was an estimated 247 million malaria cases among 3.3 billion people at risk in 2006, causing nearly a million deaths, mostly of children under five years of age. In 2008, 109 countries were reported to be endemic for malaria. Bangladesh had an estimated 2.9 million malaria cases and 15,000 deaths in 2006. Although 72% of the population are at some risk of malaria, the risk is greatest in the east and north-east of the country in areas bordering India and Myanmar. The majority of suspected cases are unconfirmed; among those that are identified as malaria, more than 70% are *Plasmodium falciparum *[1]. Malaria is a major health burden in this remote, mountainous south-eastern region of Bangladesh. Malaria transmission in Bangladesh is mostly seasonal and concentrated in the border regions with India and Myanmar. Out of the total 64 administrative districts, 13 are located along the border areas with India and Myanmar where about 98% of the total malaria morbidity and mortality reported from Bangladesh each year originate from these districts. [2]. According to passive data collected by Directorate General of Health Services (DGHS) of Bangladesh last ten years (1999-2008), the country's malaria situation remains almost steady with an annual incidence of 4% in the endemic districts.

In a recent survey, it has been found that among these 13 malaria endemic districts, the overall malaria prevalence rate was 3.1% based on Rapid Diagnostic Test (RDT). The prevalence of *P. falciparum *was 2.73% and the *Plasmodium vivax *0.16% and mixed infection with *P. falciparum *and *P. vivax *was 0.19%. The proportion of *P. falciparum *was 88.6%, while *P. vivax *and mixed infection with these two species were 5.2 and 6.25%, respectively. The overall malaria prevalence in Chittagong Hill Tracts was 11% [2].

The forests of Bangladesh have remained an area of intense malaria transmission providing a focus for re infection for the plains. Bangladesh has 34 species of anopheline mosquitoes[3]. Until 2009, only seven of these species were documented to be competent malaria vectors. Among these, four have been considered as the principal malaria vectors *i.e. Anopheles baimaii (= Anopheles dirus *D), *Anopheles philippinensis*, *Anopheles sundaicus *and *Anopheles minimus s.l. *[4]. Other species, such as *Anopheles aconitus*, *Anopheles annularis *and *Anopheles vagus*, were found to be capable of transmitting malaria during outbreak situations [5-7]. Although *An. annularis *and *An. vagus *are considered to be zoophilic, exophilic and exophagic in nature, they have been considered to maintain malaria transmission in the plain land. These two species were incriminated during epidemic situation in the flood plain areas of Bangladesh. Possibly they were incriminated due to low density of mammalian host except human [6,7].

DDT was banned in Bangladesh since 1985 and the number of malaria cases began to increase. Since then due to lack of adequate funds and programs, no control efforts maintained in the malaria endemic areas of Bangladesh[2]. Due to similar reason Malaria and Parasitic Disease Control Unit (M&PDC) of DGHS could not carry out regular entomological investigation in the endemic areas. However, they carried out sporadic entomological surveillance, but did not have the opportunity to work on incrimination of other anopheline species. Therefore, this study was designed to obtain some information regarding prevalent anopheline species and possible malaria vectors in the border belt areas where the malaria situation is endemic in Bangladesh, the results of which are presented here.

## Methods

### Study areas

**Figure 1:**
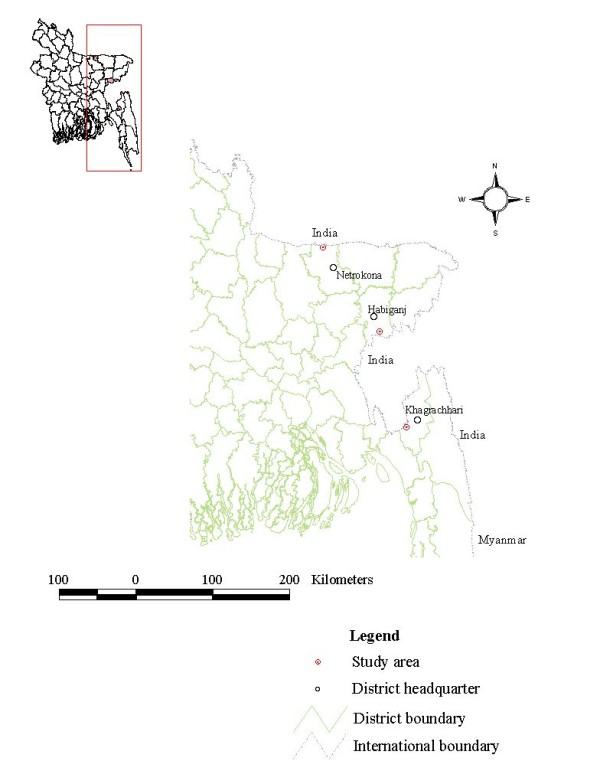
**Study areas**.

This study was conducted at three different border belt areas of Bangladesh with variable endemicity. These are Lengura (sub-district Kalmakanda: 25° 46' 0'' N, 90° 54' 0'' E) of Netrokona district, Deorgachh (including Chaklapunji tea estate; sub-district Chunarughat: 24° 11' 60" N, 91° 31' 0" E) of Habiganj district and Matiranga (sub-district Matiranga: 23° 7' 19" N, 91° 52' 36" E) of Khagrachhari district (Figure 1). Study areas were selected purposively based on the sites where DGHS conducted entomological investigation in recent past. Ecologically, Matiranga has predominately mixed thicket and dense forest and mixed evergreen and deciduous forest. Only 23% is cultivable land or fallow. Chunarughat has land cover with 51% tea plantations and 45% forest thickets interspersed with plantations like pineapple. Kalmakanda has land cover with very little forest (less than 0.1%) and is primarily agricultural. More than 77% of the land is cultivable or fallow (source: Bangladesh Bureau of Statistics).

### Collection of *Anopheles *mosquitoes

*Anopheles *mosquitoes were collected from (18.00-24.00 hours) both indoors and outdoors by human landing catches methods with the help of mouth aspirators[7]. Mosquitoes were collected from four houses per night on each of five successive nights once within the peak malaria transmission season (May to June). Four volunteers collected mosquitoes at each house two indoors and two outdoors. Every night houses was shifted randomly. Thus, in each study area for entomological surveillance, human landing catches (HLC) and resting collection were conducted in 20 households. Technical support for the entomological survey was provided by the M&PDC of DGHS.

After completion of HLC and resting collection, CDC miniature light trap model 512 (origin: Jhon W. Hock Inc, USA) was also used for entomological investigation. Each trap was installed for at least 12 hours (6 pm to 6 am). Each night four trapping was conducted for five days of a week for each of the areas alternatively once in the peak season (May to October).

### Ethical consideration

A written consent was obtained from the houses where entomological collections were made. For HLC, trained entomological technician working at DGHS were recruited. The mosquito collectors were monitored up to three months with a provision for treatment in case they got malaria, but such a case did not occur during the reported period. Ethical approvals were obtained from regional WHO research review committee and ICDDR,B ethical review committee.

### Mosquito sample preparation

The following morning after a catch, mosquitoes were sorted and identified. After identifying the species each mosquito was preserved in cryo-vial in silica gel in order to prevent microbial growth that can result in high background values.

### CSP ELISA

Circumsporozoite protein (CSP) was detected using an enzyme-linked immunosorbent assay (ELISA), as described previously [8,9]. ELISAs were used to detect *P. falciparum*, *P. vivax*-210 (VK 210), and *P. vivax*-247 (VK 247) CSP in field caught mosquitoes. *Plasmodium vivax *has two distinct polymorphs in its CSP, VK210 and VK247, that are widespread in Southeast Asia and South America[10]. In areas where the two polymorphs coexist, intrinsic biological differences between the polymorphs may affect their survival. The ratios of VK210 to VK247 were significantly higher at the end of the non-transmission season than during the annual monsoon[11]. It was also reported that fluctuations in the proportion of mosquitoes infected with the two polymorphs may reflect humoral immune pressure on the VK247 strain [12]. In each test, positive control for each *Plasmodium *species were used and for negative control field caught male *Anopheles *mosquitoes were used. Monoclonal antibody (MAB) was obtained from the Centers for Disease Control and Prevention (CDC), which were produced by Kirkegaard and Perry Laboratories (Atlanta, GA). Same batches of capture monoclonal antibodies were used in all tests. The absorbance of solution at 410 nm was determined 60 min after adding the substrate to Biorad ELISA plate reader. The cut-off was calculated by multiplying twice with the mean value of negative controls in respective tests. For ELISA positive mosquitoes tests were repeated to confirm it a positive.

## Results

A total of 634 female anopheline mosquitoes belonging to 17 species were collected by different methods (Table 1). 403 mosquitoes were collected by CDC light trap and 231 mosquitoes by other methods (HLC and resting). Majority of the mosquitoes were collected by other methods were found resting in the cattle shed, indoor or outdoor of human dwellings. *Anopheles vagus *was the dominant species (18.6%) followed by *An. nigerrimus *(14.5%) and *An. philippinensis *(11.0%). Matiranga represented with the highest number of species (15) and mosquitoes (511). Although Deorgachh and Lengura has simialr number of species (8), but mosquito numebrs were higher in Lengura (73) than Deorgachh (41). Since the numbers of mosquitoes in HLC were few, calculation for human biting rate was not performed.

**Table 1:** List of anopheline species collected from three study areas by different methods

Species		Matiranga			Deorgachh			Lengura**		Total		
	
		LT	Others*	Total	LT	Others*	Total	LT	Total	LT	Others*	Total (%)
*An. aconitus*		1	2	3	1	0	1	0	0	2	2	4 (0.6)
*An. anularis*		0	0	0	0	0	0	1	1	1	0	1 (0.2)
*An. barbirostris*		14	5	19	3	0	3	0	0	17	5	22 (3.5)
*An. jamesii*		35	0	35	0	0	0	6	6	41	0	41 (6.5)
*An. jeypurensis*		27	0	27	0	0	0	0	0	27	0	27 (4.3)
*An. karwari*		0	0	0	0	0	0	10	10	10	0	10 (1.6)
*An. kochi*		35	0	35	0	0	0	0	0	35	0	35 (5.5)
*An. maculatus*		8	0	8	0	0	0	0	0	8	0	8(1.3)
*An. minimus s.l.*		1	0	1	0	0	0	0	0	1	0	1 (0.2)
*An. niggerimus*		2	80	82	1	1	2	8	8	11	81	92 (14.5)
*An. philippinensis*		54	0	54	6	3	9	7	7	67	3	70 (11.0)
*An. subpictus*		1	58	59	0	6	6	1	1	2	64	66 (10.4)
*An. tessellatus*		10	3	13	0	0	0	0	0	10	3	13 (2.1)
*An. umbrosus*		56	1	57	5	0	5	0	0	61	1	62 (9.8)
*An. vagus*		10	60	70	5	7	12	36	36	51	67	118 (18.6)
*An. varuna*		42	2	44	0	0	0	0	0	42	2	44 (6.9)
*An. willmori*		13	0	13	0	3	3	4	4	17	3	20 (3.2)

N		309	211	520	21	20	41	73	73	403	231	634 (100)

CSP-ELISA was performed with 622 anopheline mosquitoes (remaining mosquitoes were kept as voucher specimen). 16 mosquitoes were positive in CSP-ELISA (Table 2). Thus, overall infection rate became 2.6% (16/622). Eight (1.3%) mosquitoes belonging to five species were positive for *P. falciparum*, seven (1.1%) mosquitoes belonging to five species were positive for VK210 and a single mosquito belonging to *An. maculatus *species was positive for VK 247. No mixed infection was found in this study. *P. falciparum*-positive anopheles species included one *An. barbirostris*, one *An. karwari*, four *An. vagus*, one *An. nigerrimus*, and one *An. subpictus*. VK 210 positive species included one *An. barbirostris*, one *An. karwari*, one *An. vagus*, three *An. nigerrimus*, and one *An. philippinensis*. According to species, the highest infection rate (Table 2) was observed in *An. karwari *(2/9, 22.2%) followed by *An. maculatus *(14.3%), *An. barbirostris *(9.5%), *An. nigerrimus *(4.4%), *An. vagus *(4.3%), *An. subpictus *(1.5%) and *An. philippinensis *(1.4%).

**Table 2:** CSP-ELISA positive mosquitoes Infection rate according to species

Species	No	Pf	Pv-210	Pv-247	Total
*An. aconitus*	3	0	0	0	0
*An. anularis*	1	0	0	0	0
*An. barbirostris*	21	1 (4.8)	1(4.8)	0	2 (9.5)
*An. jamesii*	41	0	0	0	0
*An. jeypurensis*	27	0	0	0	0
*An. karwari*	9	1 (11.1)	1 (11.1)	0	2 (22.22)
*An. kochi*	35	0	0	0	0
*An. maculatus*	7	0	0	1 (14.3)	1 (14.3)
*An. minimus s.l.*	1	0	0	0	0
*An. niggerimus*	91	1 (1.1)	3 (3.3)	0	4 (4.4)
*An. philippinensis*	69	0	1 (1.4)	0	1 (1.4)
*An. subpictus*	65	1 (1.5)	0	0	1 (1.5)
*An. tessellatus*	12	0	0	0	0
*An. umbrosus*	61	0	0	0	0
*An. vagus*	116	4(3.4)	1 (0.9)	0	5 (4.3)
*An. varuna*	44	0	0	0	0
*An. willmori*	19	0	0	0	0

N	622	8 (1.28)	7 (1.1)	1 (0.2)	16 (2.6)

* include HLC and resting collection, ** HLC was not done

According to place from Matiranga 11 CSP-positive (2.2%) mosquitoes had been identified in six species including *An. barbirostris, An. subpictus, An. vagus, An. nigerrimus, An. maculatus *and *An. philippinensis *(Table 3). In Lengura, five mosquitoes were identified CSP positive (6.9%) belonging to two species including *An*. *karwari *and *An*. *vagus*. In Deorgachh no mosquitoes were found CSP positive. Among 16 positive mosquitoes11 had blood on their abdomen, while seven had no visible blood meal (Table 4).

**Table 3:** Area wise CSP-ELISA positive rate

		Matiranga					Deorgachh					Lengura*		
**Species**		**LT**	**Others**	**Total**	**Positive**	**Pre (%)**	**LT**	**Others**	**Total**	**Positive**	**Pre (%)**	**LT**	**Positive**	**Pre (%)**

*An. aconitus*		1	1	2	0	0	1	0	1	0	0	0	0	-
*An. anularis*		0	0	0	-	-	0	0	0	-	-	1	0	0
*An. barbirostris*		13	5	18	2	11.1	3	0	3	0	0	0	-	-
*An. jamesii*		35	0	35	0	0	0	0	0	-	-	6	0	0
*An. jeypurensis*		27	0	27	0	0	0	0	0	-	-	0	-	-
*An. karwari*		0	0	0	-	-	0	0	0	-	-	9	2	22.2
*An. kochi*		35	0	35	0	0	0	0	0	-	-	0	-	-
*An. maculatus*		7	0	7	1	14.3	0	0	0	-	-	0	-	-
*An. minimus s.l.*		1	0	1	0	0.0	0	0	0	-	-	0	-	-
*An. niggerimus*		2	79	81	4	0.0	1	1	2	0	0	8	0	0
*An. philippinensis*		54	0	54	1	1.9	5	3	8	0	0	7	0	0
*An. subpictus*		0	58	58	1	1.7	0	6	6	-	-	1	0	0
*An. tessellatus*		9	3	12	0	0	0	0	0	-	-	0	-	-
*An. umbrosus*		55	1	56	0	0	5	0	5	0	0	0	-	-
*An. vagus*		9	59	68	2	2.9	5	7	12	0	0	36	3	8.3
*An. varuna*		42	2	44	0	0	0	0	0	-	-	0	-	-
*An. willmori*		13		13	0	0	0	2	2	-	-	4	0	0

N		303	208	511	11	2.2	20	19	39	0	0	72	5	6.9

LT: Light trap, Others: HLC and resting collection

* HLC was not done

**Table 4:** Summary table for positive anopheles female mosquitoes in CSP-ELISA from border belt areas of Bangladesh

Sample ID	Species name	Positive type	Fed*	Place of collection	Collection date	Collection type
38	*An. nigerrimus*	Pv-210	0	Matiranga	14.05.09	Others
58	*An. nigerrimus*	Pv-210	1	Matiranga	14.05.09	Others
67	*An. nigerrimus*	Pv-210	1	Matiranga	14.05.09	Others
70	*An. nigerrimus*	Pf	1	Matiranga	14.05.09	Others
134	*An. subpictus*	Pf	1	Matiranga	14.05.09	Others
185	*An. karwari*	Pf	0	Lengura	12.08.09	LT
187	*An. karwari*	Pv-210	1	Lengura	12.08.09	LT
201	*An. vagus*	Pf	1	Lengura	12.08.09	LT
209	*An. vagus*	Pf	0	Lengura	12.08.09	LT
228	*An. vagus*	Pf	1	Lengura	12.08.09	LT
264	*An. vagus*	Pv-210	1	Matiranga	14.05.09	Others
272	*An. vagus*	Pf	1	Matiranga	14.05.09	Others
314	*An. maculatus*	Pv-247	0	Matiranga	19.06.09	LT
406	*An. barbirostris*	Pf	1	Matiranga	19.06.09	LT
414	*An. barbirostris*	Pv-210	1	Matiranga	19.06.09	LT
438	*An. philippinensis*	Pv-210	0	Matiranga	19.06.09	LT

* 0 = unfed; 1 = blood fed; LT = Light trap

## Discussion

Malaria transmission pattern in Bangladesh is still poorly understood. The on-going Malaria Control Programme in Bangladesh, stresses the fact of up-to-date information on malaria vectors. As a result the current vector control programmes are being implemented on little reliable report involved in malaria transmission. Successful implementation of a vector control programme in Bangladesh, the prevalence of infection with malaria sporozoites among the local anopheline mosquitoes is important, which will help to pinpoint the main vectors and other new vectors and to develop knowledge on the bionomics of the species involved in the disease transmission.

Anopheline mosquitoes were collected from the three study sites representing three geographically different endemic regions in Bangladesh. *Anopheles vagus *and *An. philippinensis *were previously incriminated as malaria vector in Bangladesh. There was, however, no previous report in favour of infections in *An. karwari*, *An. maculatus*, *An. barbirostris*, *An. nigerrimus *and *An. subpictus *in Bangladesh.

This study was conducted within a short period of time and mosquitoes were not collected on a seasonal basis. Although it was planned to collect by similar number of trapping in all three areas but failed to do so in Deorgachh. Thus, there might be a chance to miss some of existing anopheline species there. In Lengura, the highest prevalence rate (6.9%) of CSP in Anopheles mosquitoes was found whereas in Matiranga the CSP prevalence rate was found 2.2%. No sporozoite-positive mosquito was found in Deorgachh. In Derogachh, most traps were set up or conducted HLC in Chaklapunji tea garden, a famous entomological site where bionomics of *An. baimaii *was studied in 70s [13,14] where 15 anopheline species were recorded [14]. *Anopheles baimaii*, the major vector in tea garden area was not found in this investigation. Also the numbers of anopheline species were few in the tea garden area. Three reasons could be contributing such as effects of organic pesticide (deltamethrin) for the controlling of tea plant pests, deforestation and a delay in monsoon rains in Bangladesh in 2009. Due to delay in monsoon rain and prolonged dry season natural breeding places of *An. baimaii *and other anopheline species might have disappeared [15].

The presence of CSP in some anopheline species has been reported for the first time in Bangladesh, which is an imperative finding of this study. A total of seven species was found CSP-positive in the present study. The result of this study was compared with a recent study conducted in Assam state of north-eastern India, where there was evidence of CSP infection in *An. karwari*, *An. maculatus*, *An. nigerrimus*, *An. barbirostris *and *An. subpictus *[16].

Although *An. barbirostris *and *An. subpictus *were found positive in CSP ELISA in Sri Lanka [17], they had never been incriminated as malaria vector in Bangladesh. *Anopheles vagus *was highest in this study collection and also in CSP infection (5/116, 4.3%): this species has been incriminated as malaria vector in Bangladesh [7]. Although *An. aconitus*, *An. annularis*, *An. jeyporiensis *and *An. varuna *also appeared to have vector potential [5,6,18], but CSP was not detected in these species in the present study.

CSP-ELISA has emerged as a useful tool for vector detection, indicating that several species once considered un-important in the epidemiology of malaria, such as *An. subpictus *and *An. vagus *in Sri Lanka [17]. In this study, *An. nigerrimus *was found CSP-positive, which is probably a first-time report in this region, while the species remains the principal malaria vector in the Indo-Chinese Hills and the Malaysian Zones(Varma MG: Geographical distribution of arthropod borne disease and their principal vectors, unpublished document WHO/VBC/89967). Similarly, *An. karwari *is considered a secondary vector in the Australian region [19], but its vectorial status in South-East Asia was unknown. There is still remaining controversy for CSP-ELISA particularly due to its false positive results in previous studies. Thus, positivity in a CSP-ELISA should not be taken as the only criterion in confirming the vector status of an *Anopheles *species [20-22].

The present result does not report any infection evidence in *An. minimus s.l. *This might be due to only one mosquito of this species was tested. In a recent study conducted in Chakaria of Bangladesh, which is geographically similar to Matiranga, a higher percentage of *An. minimus s.l. *was caught (97.3%; 651/669), of which 19 were positive for *Plasmodium *infection by the microtiter plate hybridization (MPH) method[23]. Before 1950, *An. minimus s.l. *was the principal vector recognized in Bangladesh, but its population declined due to the routine spraying of DDT, to which it remains susceptible[14]. In recent past, the density of *An. minimus s.l. *was negligible, as observed in a few sporadic entomological investigations carried out by M&PDC (personal communication with NP Maheswary, a veteran entomologist). Hence, the higher number of *An. minimus s.l. *reported in Chakaria might be due to misidentification. A similar situation occurred in Vietnam where formally identified *An. minimus s.l. *was found to be *Anopheles varuna *[24]. A reasonable number of *An. varuna *in the present study is also supportive to this fact.

## Conclusions

Findings of this study suggested that anopheline species other than *An. minimus s.l. *and *An. baimaii *might have a role in the transmission of malaria in endemic areas of Bangladesh. The detection of CSP in some anopheline species should be taken into consideration for further studies to investigate their possible role in malaria transmission in Bangladesh.

## Competing interests

The authors declare that they have no competing interests.

## Authors' contributions

MSA conceptualized and designed the study collected and identified sample, analyzed data, drafted the manuscript and made final revisions. MGMK, SD, FN did sample analysis and made critical revision of the manuscript. NC organized the field activities, analysed data and helped in the revision of the manuscript. AMB and RH participated in study design, critical analysis of data and helped in drafting the manuscript. All authors read the final manuscript and approved.
